# Incidental Atrial Myxoma in the Shadow of COVID-19: Coincidence or an Emerging Pattern?

**DOI:** 10.7759/cureus.93617

**Published:** 2025-09-30

**Authors:** Bilal Rashid, Isfandyar Baig, Muhammad Dawar Feroze, Manahil Chaudhry, Muhammad Sardar Zeeshan Hassan

**Affiliations:** 1 Acute Medicine, Lancashire Teaching Hospitals National Health Service (NHS) Foundation Trust, Preston, GBR; 2 General Practice, Mersey and West Lancashire Teaching Hospitals National Health Service (NHS) Trust, Preston, GBR; 3 General Surgery, Shalamar Medical and Dental College, Lahore, PAK; 4 Emergency Medicine, Combined Military Hospital, Lahore, PAK

**Keywords:** cardiac endothelitis, cardiac tumour, covid-19, left atrial myxoma, silent myxoma

## Abstract

Primary cardiac tumours are a rare entity, and atrial myxomas are a benign subtype usually found in the left atrium. They can present with a wide array of symptoms ranging from cardiac complications to systemic emboli. On the contrary, they can also manifest silently and are detected incidentally, as in this case of an 83-year-old female patient. New lung changes on an X-ray done as a part of an infection screen ultimately led to the diagnosis of an atrial myxoma confirmed by a transthoracic echocardiogram (TTE) and a transoesophageal echocardiogram (TOE). The patient was commenced on anticoagulation and was referred for cardiac surgery. Notably, she had suffered a COVID-19 infection recently, which may have acted as a catalyst in the formation of the tumour. Although a direct causal relationship cannot be established, the literature suggests that COVID-19 infection may lead to chronic inflammation, immune dysregulation, endothelial dysfunction, and other direct viral effects on tissues, all of which could potentially contribute to tumour formation.

## Introduction

Cardiac neoplasms can be broadly classified into primary (arising de novo) or secondary (metastatic). Primary tumors form only 0.3-0.7% of the entire tumour burden [1]. The most commonly occurring among them is the atrial myxoma [2]. It is a soft, gelatinous growth attached to the upper cardiac chambers, usually favouring the left atrium. Although benign, it presents with a wide array of symptoms caused by the obstruction of the mitral or tricuspid valve, constitutional symptoms such as fever and weight loss, fragmentation of the tumour resulting in embolic phenomena and the worst-case outcome, sudden death [3-5]. Atrial myxomas are rarely silent. They largely arise sporadically with only 7% being familial in origin [4,6,7]. In this case, there was sudden sighting of the myxoma in an elderly female patient shortly after she tested positive for COVID-19. We aim to draw attention to the severe acute respiratory syndrome coronavirus 2 (SARS-CoV-2) infection potentially being linked to the formation or progression of an atrial myxoma.

## Case presentation

An 83-year-old, White British female patient was admitted to the acute medicine department after presenting with fever, watery stools, reduced oral intake and lethargy for the previous eight days. She reported being COVID-19 positive two months prior on home testing after she felt generally unwell with myalgias and a fever, post-contact with an infected family member. She had previously been vaccinated with four doses of the mRNA COVID-19 vaccine. Her past medical and surgical history included a left shoulder joint replacement and seropositive rheumatoid arthritis (RA).

**Figure 1:**
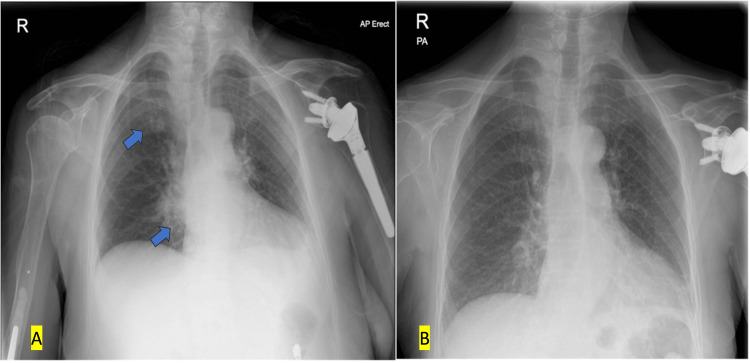
A: chest x-ray on current admission showing a nodule in the right upper zone with a bulky right hilum (blue arrows). B: old chest x-ray for comparison taken approximately one year ago. Panel A: New right-sided lung opacities highlighted by blue arrows. Panel B: Old chest x-ray showing showing absence of any abnormal lung findings.

Her vitals on admission were stable, and labs showed a raised C-reactive protein (CRP) of 141.2 mg/L and neutrophilic leucocytosis. A rapid gastrointestinal panel was positive indicating acute gastroenteritis. She also had a chest X-ray on this admission as part of the infection screen (Figure 1A), which showed new changes when compared to her previous chest X-ray (Figure 1B) taken a year ago.

She was managed with supportive therapy and a positive clinical response over the next 48 hours was noted. The CRP dropped to 58 mg/L. She was discharged with a follow-up computed tomography (CT) scan of the chest arranged to further evaluate the new right-sided lung findings. The scan was done within the next two weeks.

**Figure 2:**
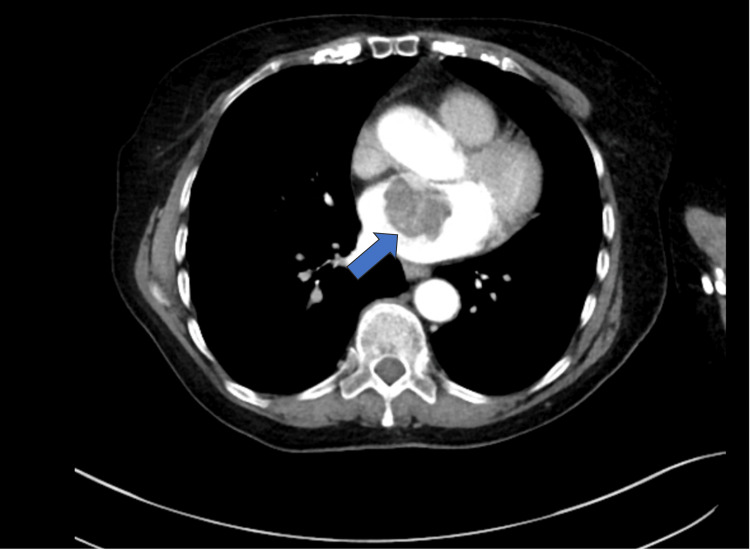
Chest computed tomography (CT) scan showing a heterogeneous mass within the left atrium (blue arrow) measuring 3.9x3.8x4.3 cm (anteroposterior, transverse, craniocaudal).

On outpatient review, the radiology team noted a heterogeneous, non-obstructing mass, possibly a myxoma, within the left atrium projecting from the anterior atrial wall (Figure 2). Given the findings they prompted an elective admission under the acute medicine team for monitoring and further evaluation.

She was re-admitted within a week from the CT scan and was asymptomatic on presentation. She denied any shortness of breath, chest pain, dizziness, or palpitations. Systemic examination was unremarkable, including no audible murmurs. Her electrocardiogram (ECG) showed normal sinus rhythm with a normal axis and no acute features. A cardiology consult was sought and the process of evaluating the new mass was commenced in the form of a transthoracic echocardiogram (TTE) (Video 1), which was done the same day. This confirmed the presence of a rounded, jelly-like, non-mobile echogenic mass within the left atrium with a possible pedicle attached close to the intra-atrial septum. These findings were consistent with an atrial myxoma. The measurements were as follows: 3.8 cmx3.3 cm with an area of 13 cm^2^. All valves were functionally and morphologically normal. The left atrial appendage was clean, and the intra-atrial septum was intact with no patent foramen ovale. A transoesophageal echocardiogram (TOE) was also performed (Video 2) within two days of the TTE which confirmed the diagnosis of a left atrial myxoma.

**Video 1:** Transthoracic echocardiogram (TTE) - apical long axis view showing a fixed mass within the left atrium causing no obstruction of flow accross the mitral valve.

**Video 2:** Transoesophageal echocardiogram (TOE) showing a non-mobile, well-circumscribed mass in the interior of the left atrium. TOE reveals a large atrial myxoma, which is moving minimally with cardiac cycles.

She was referred to the cardiothoracic team for its surgical removal. Anticoagulation was initiated while awaiting surgery to mitigate the embolization risk. A direct oral anticoagulant (DOAC), apixaban, was commenced due to its rapid onset of action and predictable anticoagulant effect without the need for routine international normalised ratio (INR) monitoring.

## Discussion

Sporadically occurring myxomas are more common in women and are usually detected between the third and sixth decades of life [8]. In this case study, we observed atrial myxoma presenting in an 83-year-old female patient. The tumour was asymptomatic and hence discovered incidentally during diagnostic workup for infection. Generally, the location within the heart, size, mobility, and nature of the tumour - whether smooth, friable, or villous - determines the resulting symptoms, where friable and villous forms pose a higher risk of embolisation [9]. Nasution et al. reported a patient suffering from COVID-19 in whom a prolapsing left atrial myxoma resulted in an ischemic stroke [2]. Our patient had a smooth, non-mobile mass, which can explain why it manifested silently. The size of the myxoma commonly ranges from 2 to 6 cm, and in our case, the myxoma measured around 4 cm in the largest dimension. However, an atrial myxoma as large as 20 cm has been reported by Shakerian et al. in a patient with concurrent COVID-19 infection [10].

Atrial myxoma can be detected on various forms of imaging and can be similar in appearance to a cardiac thrombus, or other benign cardiac tumours such as a fibroelastoma, lipoma, or rhabdomyoma, malignant tumours and physiological structures such as crista terminalis (a muscular ridge) or the Coumadin ridge (left atrial tissue). Discerning features of an atrial myxoma are its heterogeneous appearance and a pedicle attachment in the atria, both of which were seen in this case on chest and cardiac imaging and confirmed the diagnosis. The imaging techniques with high sensitivity and specificity for diagnosis of the tumour are the TTE and TOE with TOE being superior, which has a reported sensitivity of 100% [11]. In terms of treatment, surgical resection is the only curative option with early intervention being fundamental to prevent cardiovascular complications. 

We suspect that in our patient the tumour had developed rapidly, confirmed by its absence in the patient’s former TTE done approximately one year ago. Alrifae et al. reported a case of a myxoma associated with a thrombus in a post-acute COVID-19 syndrome (PACS) patient who was suspected to have the infection four months ago [12]. In our case, the patient suffered from a mild to moderate COVID-19 infection two months prior to the discovery of the atrial myxoma. 

We now direct our attention to the possible mechanisms of how the COVID-19 virus can result in this. The viral components bind to the cardiac cells via a spike protein, leading to endothelial inflammation [12]. Mitrofanova et al. published a study showing immunohistochemical evidence of the severe acute respiratory syndrome coronavirus 2 (SARS-CoV-2) spike protein dwelling in the cardiac tumour cells, which although is only preliminary evidence and does not establish a direct relationship but plausibly explains the association [13]. In addition, the inflammatory reaction in synergy with the ongoing cytokine storm due to the host-immune response can promote tumour formation. The SARS-CoV-2 components have also been shown to alter cellular DNA and its repair mechanisms, which are pathways in carcinogenesis [14]. Overall, a post-pandemic statistically significant increase in myxoma cases (1.5-fold) has also been reported [13]. These accounts, along with this case, are sufficient to warrant larger epidemiological studies to confirm this evolving trend.

## Conclusions

Atrial myxomas are a rare entity among the intra-cardiac tumours. We present the case of an asymptomatic atrial myxoma, which we assume is consequential to a recent mild COVID-19 episode. Since the pandemic, a possible association between COVID-19 and a rise in the incidence of cardiac tumours, notably myxomas has been recognised. This hypothesised link warrants further study to add to our understanding of the cardiac manifestations of SARS-CoV-2.
